# Dietary Cholesterol Contained in Whole Eggs Is Not Well Absorbed and Does Not Acutely Affect Plasma Total Cholesterol Concentration in Men and Women: Results from 2 Randomized Controlled Crossover Studies

**DOI:** 10.3390/nu10091272

**Published:** 2018-09-09

**Authors:** Jung Eun Kim, Wayne W. Campbell

**Affiliations:** 1 Food Science & Technology Programme, c/o Department of Chemistry, National University of Singapore, Science Drive 2, Singapore 117546, Singapore; 2 Department of Nutrition Science, Purdue University, 700 W State Street, West Lafayette, IN 47907, USA

**Keywords:** whole egg, dietary cholesterol, triacylglycerol-rich lipoprotein fractions, total cholesterol, triacylglycerol

## Abstract

Whole egg is a food source of dietary cholesterol and inconsistent research findings exist about the effect of dietary cholesterol from whole egg on blood cholesterol concentration. We assessed the effect of co-consuming cooked whole egg (CWE) on dietary cholesterol absorption from two randomized-crossover studies. For study 1, 16 men consumed raw vegetables with no egg, 75 g CWE, or 150 g CWE. For study 2, 17 women consumed cooked vegetables with no egg or 100 g CWE. Triacylglycerol-rich lipoprotein fractions (TRL) were isolated from collected blood. In study 1, total-cholesterol areas under the curve (AUC)_0–10h_ in TRL were not different but triacylglycerol AUC_0–10h_ in TRL was greater for 150 g CWE vs. 75 g CWE and no egg. Similarly, in study 2, total-cholesterol AUC_0–10h_ in TRL was not different but triacylglycerol AUC_0–10h_ in TRL was greater for 100 g CWE vs. no egg. In both studies, whole egg consumption did not affect plasma total-cholesterol AUC_0–10h_, while triacylglycerol AUC_0–10h_ was increased. These results suggest that the dietary cholesterol in whole egg was not well absorbed, which may provide mechanistic insight for why it does not acutely influence plasma total-cholesterol concentration and is not associated with longer-term plasma cholesterol control.

## 1. Introduction

Cholesterol’s important functions in the human body include an essential structural component in cell membranes and being a chemical precursor of steroid hormones and bile acids [1,2]. Cholesterol is also a major component of the human brain [3]. Although about 15–25% of total body cholesterol comes from diet [4], dietary cholesterol is implicated in increasing blood total and low density lipoprotein (LDL) cholesterol concentrations [5], and the risk of cardiovascular diseases (CVD) [6,7]. Previous Dietary Guidelines for Americans recommended for the general population of the United States to consume no more than 300 mg/day of dietary cholesterol [8,9]. However, research presented in the 2015 Dietary Guidelines Advisory Committee report [10] and the 2013 American College of Cardiology/American Heart Association Lifestyle Guideline for the Reduction of Cardiovascular Disease [11] brought into question the apparent association between dietary cholesterol consumption and blood cholesterol concentration, thus negating the recommendation for Americans to limit dietary cholesterol intake.

Whole egg is a well-known food source of dietary cholesterol and contains unsaturated fat, high-quality protein, and varying quantities of vitamins and minerals [12,13]. Despite the nutritional benefits, egg consumption is generally discouraged due to the high cholesterol content in egg yolk [14]. However, inconsistent research findings exist about the effect of dietary cholesterol from whole egg on CVD risk [15,16,17] and favorable changes in blood lipids and lipoproteins after whole egg consumption are suggested [18]. Although consumption of increasing amounts of dietary cholesterol increased postprandial plasma phospholipid concentration and triacylglycerol content within triacylglycerol-rich lipoprotein (TRL) fractions [19], the absorption of dietary cholesterol from whole egg in humans requires investigation. Specifically, postprandial changes in total cholesterol concentration within TRL fractions, which includes biliary cholesterol and newly absorbed cholesterol from a consumed meal [4,20], require investigation.

The main objective of this research was to assess the effect of consuming cooked whole eggs on cholesterol absorption in adults, measured in TRL fractions. Triacylglycerol concentration within TRL fractions was also measured and used as a control comparison. The results presented in this manuscript were obtained using secondary data from two randomized, cross-over, controlled acute feeding studies designed to assess the impact of consuming eggs on the absorption of fat soluble nutrients from a co-consumed meal [21,22].

## 2. Materials and Methods

### 2.1. Studies 1 and 2: Ethics and Protocol Registration

The Purdue University Biomedical Institutional Review Board approved both studies. All subjects signed an informed consent form and received monetary compensation for their participation. Clinical trial profiles for study 1 and study 2 are described at NCT01951313 and NCT02679794, respectively.

### 2.2. Study 1: Subjects

**Figure 1:**
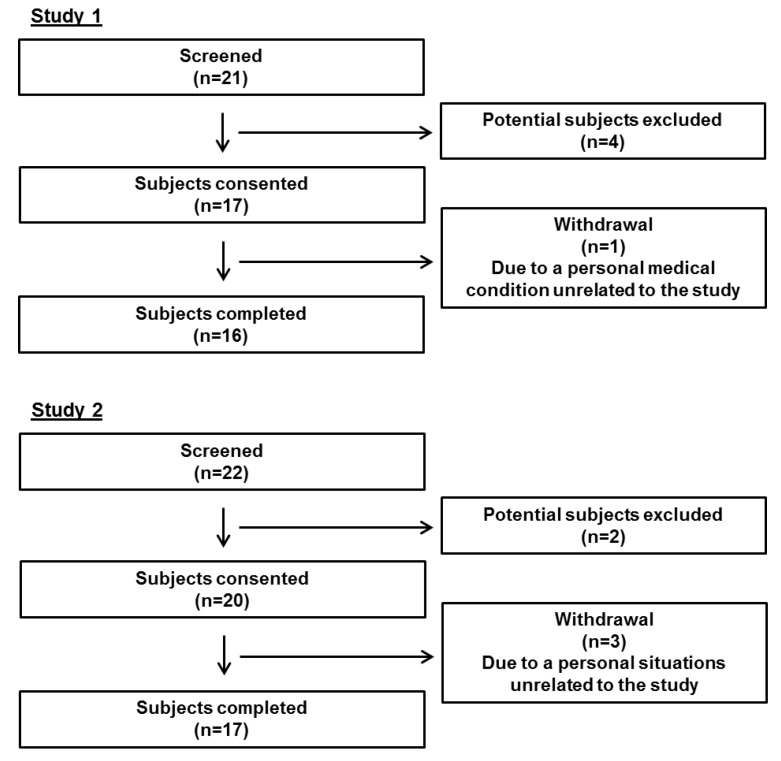
Consolidated Standards of Reporting Trials flow diagrams for study 1 and study 2.

Sixteen men from the greater Lafayette, IN, region completed the study (Figure 1). Inclusion criteria included weight stability (±3 kg in the past 3 months); no vigorous exercising over the past 3 months; no intestinal disorders including fat mal-absorption or lactose intolerance; normal liver and kidney functions; fasting blood glucose <110 mg/dL; no smoking; not drinking more than 2 alcoholic beverages per day; and not taking lipid-lowering medications or dietary supplements affecting plasma cholesterol concentration.

### 2.3. Study 1: Study Design

For this single-blinded, randomized and crossover-design study [22], all subjects completed 3 trials; SAS 9.2 software (SAS Institute Inc., Cary, NC, USA) was used to randomize the trial orders. The investigators were blinded with regard to treatment order until all subjects finished the protocol and all sample analyses were completed, while the subjects and dietitians were not blinded. Prior to each testing day, subjects consumed a prescribed low carotenoid diet for 7-day. On each of the three testing days, subjects came to the Purdue clinical research center after a 12-h overnight period of fasting and a catheter was placed into an antecubital vein. After a baseline blood sample was collected, subjects consumed a test meal based on their randomization order. After the test meal was consumed, blood samples were collected hourly for 10 h and lunch was consumed after blood was collected at hour 5. The 3 periods of prescribed diet and testing were each separated by one-week dietary washout periods when subjects consumed their habitual unrestricted diets.

### 2.4. Study 1: Test Meal

On each of the three testing days, subjects consumed a raw mixed-vegetable salad (0 mg dietary cholesterol) including tomatoes, shredded carrots, baby spinach, lettuce, and Chinese wolfberry with no egg, with 75 g cooked whole egg (CWE), or with 150 g CWE. All salads were also served with 3 g canola oil. A served raw mixed-vegetable salad with 3 g canola oil provided 92 kcal, 3 g protein, 12 g carbohydrate, 4 g fat, and 0 mg of cholesterol. The no egg, 75 g CWE, and 150 g CWE included 0 mg, 280 mg, and 560 mg of total cholesterol, respectively. Cooked whole eggs were prepared uniformly from large eggs based on the American Egg Board’s recommendation [23] and portioned appropriately to dose. The low-cholesterol lunch consumed at hour 5 provided 526 kcal, 23 g protein, 100 g carbohydrates, 4 g fat, and 19 mg of cholesterol. All menus were developed by a registered dietitian using Pronutra software version 3.3 (Viocare, Inc. Princeton, NJ, USA) and all foods were prepared, portioned, and provided to the subjects by research staff in the Department of Nutrition Science Metabolic Kitchen at Purdue University.

### 2.5. Study 2: Subjects

Seventeen women from the greater Lafayette, IN, region completed the study (Figure 1). Inclusion criteria included weight stability (±3 kg in the past 3 months); no vigorous exercising over the past 3 months; no intestinal disorders including fat mal-absorption or lactose intolerance; normal liver and kidney functions; blood 25-hydroxyvitamin D ≥20 nmol/L; fasting blood glucose <110 mg/dL; no smoking; not drinking more than 2 alcoholic beverages per day; not taking estrogen-based birth control or osteoporosis prevention or treatment medications in the past 3 months; and not taking lipid-lowering medications or dietary supplements affecting plasma cholesterol concentration.

### 2.6. Study 2: Study Design

For this investigator-blinded, randomized, crossover-design study SAS 9.2 software (SAS Institute Inc., Cary, NC, USA) was used to randomize the trial orders and all subjects completed the two trials. Prior to each testing day, subjects consumed a prescribed low carotenoid and low-vitamin D diet for 7-day. On each of the two testing days, a catheter was placed into an antecubital vein and after a baseline blood sample was collected, subjects consumed a test meal. After the test meal was consumed, blood samples were collected hourly for 10 h and lunch was consumed after blood was collected at hour 5. Three-week dietary washout periods were scheduled between the 2 prescribed diet and testing periods since menstrual cycle phase may affect lipid and lipoprotein metabolism [23,24].

### 2.7. Study 2: Test Meal

On each of the two testing days, subjects consumed sautéed vegetables (0 mg dietary cholesterol) including carrots, spinach, pepper, tomato salsa, and vitamin D enriched Portobello mushroom and 3 g canola oil with no egg or with 100 g CWE. The served sautéed vegetables with 3 g canola oil provided 92 kcal, 3 g protein, 12 g carbohydrate, 4 g fat, and 0 mg of cholesterol. The no egg and 100 g CWE included 0 mg and 373 mg of total dietary cholesterol, respectively. The low cholesterol lunch consumed at hour 5 provided 443 kcal, 15 g protein, 88 g carbohydrates, 3 g fat, and 14 mg of cholesterol. A registered dietitian developed all menus, as described above.

### 2.8. Studies 1 and 2: Sample Collection and Analyses

#### 2.8.1. Baseline Blood Sample Collection and Lipid-Lipoprotein Analysis

Fasting state bloods were collected into serum separator tubes and tubes were held at room temperature for 30 min and centrifuged at 4000× *g* at 4 °C for 15 min. Serum tubes were sent to MidAmerica Clinical Laboratories (Indianapolis, IN, USA) and lipid-lipoprotein profiles (triacylglycerol, total cholesterol, and high density lipoprotein (HDL) cholesterol) were measured using photometric assays (Chemistry Immuno Analyzer AU5700; Olympus, Center Valley, PA, USA) and low density lipoprotein (LDL) cholesterol was calculated using the Friedewald equation [25].

#### 2.8.2. Blood Sample Collection and Plasma and Triacylglycerol-Rich Lipoprotein Fraction Isolation

On each testing day, bloods were collected into EDTA tubes and portions of collected blood samples were centrifuged (3000× *g*, 15 min, 4 °C) to obtain plasma and aliquots of plasma were stored at −80 °C until thawed for analyses. Ten mL of fresh plasma were also processed to isolate the TRL fractions as previously reported [21,22]. The isolated TRL fractions were pipetted into cryo-storage tubes, which were flushed with nitrogen gas and stored at −80 °C until thawed for analysis.

#### 2.8.3. Total Cholesterol and Triacylglycerol Analyses

Total cholesterol and triacylglycerol concentrations within the TRL fractions and plasma were assessed in duplicate using a Cobas MIRAS Plus chemistry analyzer (Roche Analytical Instruments, Nutley, NJ, USA). Postprandial total cholesterol and triacylglycerol concentrations within the TRL fractions and plasma were baseline corrected by subtracting fasting concentrations from each time point. The 0–10 h positive incremental areas under the curve (AUC) of total cholesterol and triacylglycerol concentrations in TRL fractions were then calculated.

### 2.9. Studies 1 and 2: Power Calculation and Statistical Analysis

Since the assessment of whole egg consumption on cholesterol absorption is a secondary objective of both studies, subject sample size estimates were not done based on this outcome of interest. Retrospectively, we conducted effect-size calculations before implementing the data analysis. For these within-subject, crossover-designed studies, an a priori power calculation was completed for two dependent means (no egg vs. 150 g CWE for study 1 and no egg vs. 100 g CWE for study 2, correlation = 0.5) to detect a difference equal to 1 SD between treatments (a = 0.05; 90% power; 2 tailed). The effect size was one and the total number of participants needed for each study was estimated to be 13, which is less than the 16 subjects tested for Study 1 and 17 subjects tested for Study 2.

Age and BMI adjusted repeated-measures ANOVA with post hoc Tukey’s test was performed to determine differences of baseline corrected total cholesterol and triacylglycerol concentrations at each time point. Age and BMI adjusted one-factor ANOVA with post hoc Tukey’s test was also applied to assess the differences in baseline-corrected positive incremental AUC_0–10h_ of total cholesterol and triacylglycerol in TRLs and plasma. All of the analyses were performed using SAS 9.2 (SAS Institute Inc., Cary, NC, USA) and data are presented as least-squares means (lsmeans) ± standard error (SE) of the lsmean unless otherwise noted. Statistical significance was accepted at *p* < 0.05 (2-tailed).

## 3. Results

### 3.1. Subject Baseline Characteristics

#### 3.1.1. Study 1

The mean ± SE age and BMI of the 16 men were 24 ± 1 year and 24 ± 1 kg/m^2^, respectively. Blood lipid and lipoprotein concentrations were 99 ± 14 (triacylglycerol), 171 ± 8 (total cholesterol), 52 ± 3 (HDL cholesterol), and 100 ± 7 (LDL cholesterol) mg/dL.

#### 3.1.2. Study 2

Among the 17 women, mean ± SE age was 45 ± 4 year and BMI was 25 ± 2 kg/m^2^. Blood lipid and lipoprotein concentrations were as follows: triacylglycerol, 90 ± 12; total cholesterol, 183 ± 11; HDL cholesterol, 56 ± 3; and LDL cholesterol, 109 ± 10 mg/dL.

### 3.2. Total Cholesterol and Triacylglycerol Concentrations in TRL Fractions

#### 3.2.1. Study 1

**Figure 2:**
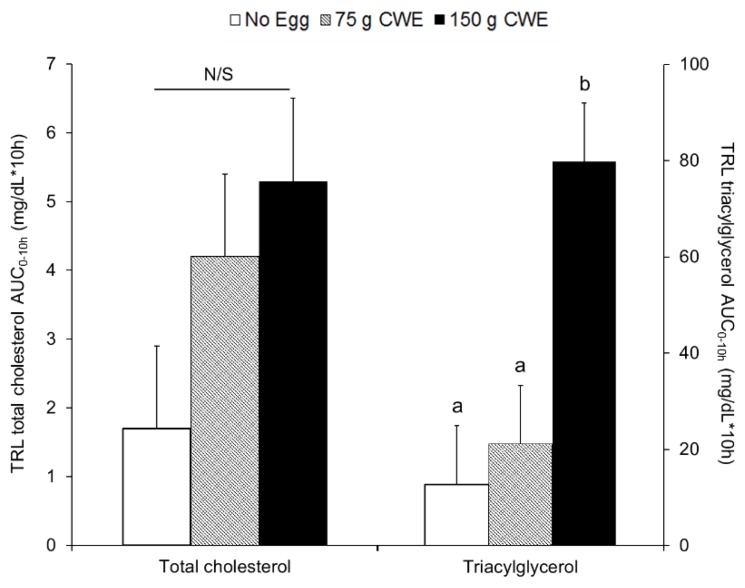
Total cholesterol and triacylglycerol AUC_0–10h_ within TRL fractions in study 1. Values are lsmean ± SE and different superscript letters (a, b) indicate statistical differences among no egg, 75 g CWE, and 150 g CWE (*p* = 0.0006). AUC, areas under the curve; CWE, cooked whole egg; N/S, not significant; TRL, triacylglycerol-rich lipoprotein.

Total cholesterol concentrations within TRL fractions at each time point were not different among trials during the 10 h of testing (Supplemental Figure S1 A). The total cholesterol AUC_0–10h_ within TRL fractions also were not different among 150 g CWE vs. 75 g CWE vs. no egg (lsmean ± SE; 5.3 ± 1.2 vs. 4.2 ± 1.2 vs. 1.7 ± 1.2 mg·dL^−1^·10 h, *p* = 0.10) (Figure 2). In contrast, from hours 3 to 6, the 150 g CWE presented greater triacylglycerol concentrations within TRL fractions than did no egg (Supplemental Figure S1B). The triacylglycerol AUC_0–10h_ within TRL fractions was greater for 150 g CWE vs. 75 g CWE and no egg (80 ± 12b vs. 21 ± 12a vs. 13 ± 12a mg·dL^−1^·10 h, *p* = 0.0006) (Figure 2).

#### 3.2.2. Study 2

**Figure 3:**
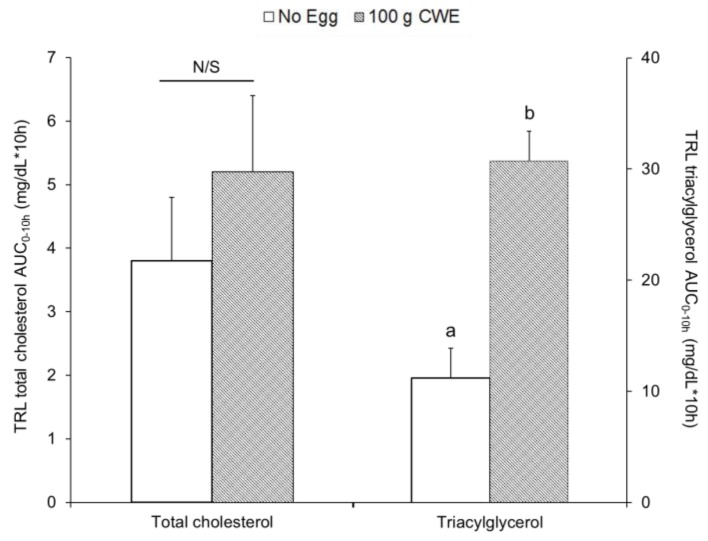
Total cholesterol and triacylglycerol AUC_0–10h_ within TRL fractions in study 2. Values are lsmean ± SE and different superscript letters (a, b) indicate statistical differences between no egg and 100 g CWE (*p* < 0.0001). AUC, areas under the curve; CWE, cooked whole egg; N/S, not significant; TRL, triacylglycerol-rich lipoprotein.

Similar to study 1, total cholesterol concentrations within TRL fraction during the 10 h of testing (Supplemental Figure S2A) and the total cholesterol AUC_0–10h_ within TRL fractions were not different between 100 g CWE vs. no egg (5.2 ± 1.0 vs. 3.8 ± 1.0 mg·dL^−1^·10 h, *p* = 0.30) (Figure 3). At hours 3 and 4, the 100 g CWE treatment presented a greater triacylglycerol concentrations within TRL fractions than no egg (Supplemental Figure S2B) and the triacylglycerol AUC_0–10h_ within TRL fractions was greater for 100 g CWE vs. no egg (31 ± 3b vs. 11 ± 3a mg·dL^−1^·10 h, *p* < 0.0001) (Figure 3).

### 3.3. Total Cholesterol and Triacylglycerol Concentrations in Plasma

#### 3.3.1. Study 1

**Figure 4:**
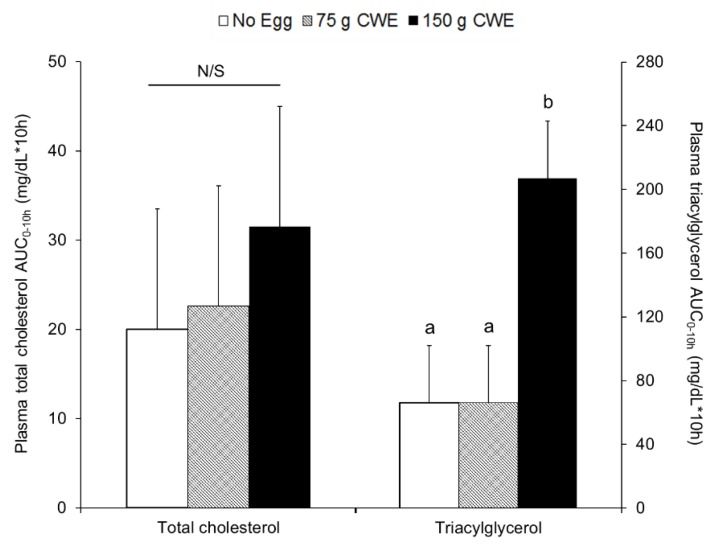
Total cholesterol and triacylglycerol AUC_0–10h_ within plasma in study 1. Values are lsmean ± SE and different superscript letters (a, b) indicate statistical differences among no egg, 75 g CWE, and 150 g CWE treatments (*p* = 0.0099). AUC, areas under the curve; CWE, cooked whole egg; N/S, not significant.

Plasma total cholesterol concentrations during the 10 h of testing (Supplemental Figure S3) and the plasma total cholesterol AUC_0–10h_ were not different among 150 g CWE vs. 75 g CWE vs. no egg (lsmean ± SE; 32 ± 14 vs. 20 ± 14 vs. 22 ± 14 mg·dL^−1^·10 h, *p* = 0.83) (Figure 4). From hours 3 to 6, the 150 g CWE treatment presented a greater plasma triacylglycerol concentrations than did 75 g CWE and no egg (Supplemental Figure S3) and the plasma triacylglycerol AUC_0–10h_ was greater for 150 g CWE vs. 75 g CWE and no egg (207 ± 36b vs. 66 ± 36a vs. 66 ± 36a mg·dL^−1^·10 h, *p* = 0.0099) (Figure 4).

#### 3.3.2. Study 2

**Figure 5:**
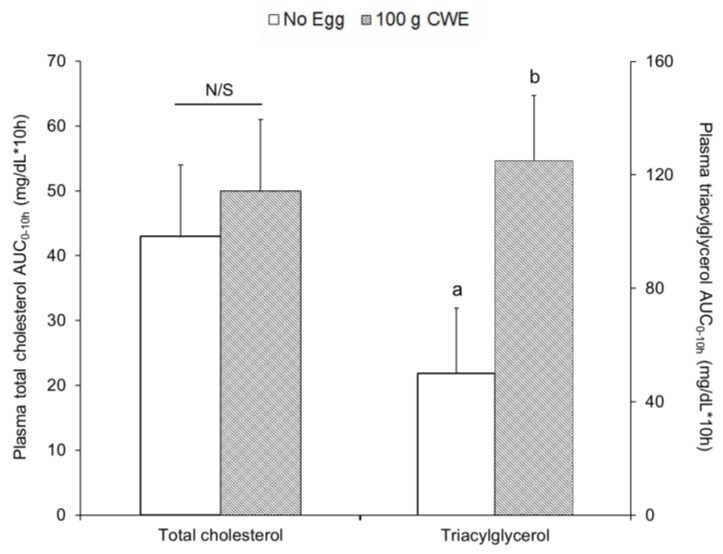
Total cholesterol and triacylglycerol AUC_0–10h_ within plasma in study 2. Values are lsmean ± SE and different superscript letters (a, b) indicate statistical differences between no egg and 100 g CWE treatments (*p* = 0.0281). AUC, areas under the curve; CWE, cooked whole egg; N/S, not significant.

Similarly, plasma total cholesterol concentrations during the 10 h of testing (Supplemental Figure S4) and the plasma total cholesterol AUC_0–10h_ were not different between 100 g CWE vs. no egg (50 ± 11 vs. 43 ± 11 mg·dL^−1^·10 h, *p* = 0.66) (Figure 5). From hours 3 to 6, the 100 g CWE presented greater plasma triacylglycerol concentrations than no egg (Supplemental Figure S4) and the plasma triacylglycerol AUC_0–10h_ was greater for 100 g CWE vs. no egg (125 ± 23b vs. 50 ± 23a mg·dL^−1^·10 h, *p* = 0.0281) (Figure 5).

## 4. Discussion

Historically, consumers were discouraged from consuming whole egg due to the relatively high content of dietary cholesterol in egg yolk. Yet, previous studies showed no relation between the whole egg intake and blood cholesterol concentration [26,27,28]. Several scientific researchers suggest a reconsideration of recommendations to limit dietary cholesterol and egg cholesterol consumption due to conflicting evidence [29,30]. The findings from the current two studies indicate that the dietary cholesterol found in whole eggs may not be well absorbed and does not acutely affect plasma total cholesterol concentration.

While fasting state lipid and lipoprotein concentrations mainly reflect body homeostasis, their post-prandial responses reflect the capacity to handle an acute dietary fat and cholesterol load [31]. Experimentally, more accurate assessments of dietary cholesterol absorption may be made by measuring changes in cholesterol concentrations in TRL fractions versus plasma because changes in cholesterol in TRL fractions may represent newly absorbed dietary cholesterol [4,19,20]. Using the TRL technique, our results of limited dietary cholesterol absorption from whole eggs in apparently healthy young men (Study 1) and young and middle-aged women (Study 2) are consistent with results from healthy men who consumed different amounts of dietary cholesterol (0, 142, 284, and 710 mg) from cooked egg yolk [19]. The presence of phosphatidylcholine and sphingomyelin in egg yolk may partially explain these observations. Ingestion of these phospholipids influence intestinal lipid metabolism [32] and decreased lymphatic absorption of cholesterol [33,34]. Egg white protein may also help reduce cholesterol absorption by inhibiting micellar solubility of cholesterol in the intestine [35]. Along with a limited dietary cholesterol absorption, plasma total cholesterol concentration was not affected by increasing whole egg intake in this study and previous study also found no effect of whole egg consumption (0-, 1-, 2-, and 4-whole egg diet) on postprandial total cholesterol concentration in healthy young men [36]. In contrast, healthy men who consumed increasing amount of dietary cholesterol from egg yolk presented greater postprandial plasma cholesterol concentration [19]. Research with rats showed that a whole egg-enriched diet lowered plasma LDL concentration and increased fecal bile acid content, compared to a high-cholesterol diet and egg yolk-enriched diet (dietary cholesterol content matched) [37]. The rats fed the whole egg-enriched diet had higher mRNA levels of LDL-receptor and cholesterol 7a hydroxylase, consistent with whole egg activating LDL receptor–mediated catabolism, bile acid synthesis, and the excretion of fecal cholesterol. Egg white protein is suspected to have a favorable effect on blood lipoprotein profiles [38] and limited research in humans observed that egg white protein ingestion reduced serum cholesterol in young women [39]. In the current study, phosphatidylcholine and sphingomyelin from egg yolk and egg white protein may have contributed to limiting cholesterol absorption after subjects consumed whole eggs.

While results from both study 1 and study 2 indicate that consuming cholesterol-rich whole eggs did not acutely increase cholesterol absorption or plasma total cholesterol concentrations, higher triacylglycerol concentrations within the TRL fractions and plasma were observed. These results are consistent with research showing that higher dietary fat intake (0 g vs. 45 g) caused greater triacylglycerol within TRL fractions and plasma [19], and that progressively higher dietary fat content of meals increased postprandial plasma triacylglycerol responses [31]. Another previous human intervention study also assessed the impact of amount (3, 8, and 20 g) and source (canola oil, soybean oil, or butter) of dietary fat on postprandial triacylglycerol responses and regardless of source, consuming higher amount of dietary fat induced greater triacylglycerol content within TRL fractions [40]. This greater absorption of triacylglycerol may explain the greater absorption of carotenoids and vitamin E from the mixed vegetable meals consumed by our participants [21,22] since those nutrients are fat-soluble and co-consuming dietary fat enhances their absorption [40,41,42]. Collectively, although cholesterol absorption may be limited with whole egg intake, they may not affect the absorption of triacylglycerol and fat-soluble nutrients.

Strengths of this research include using data from two investigator-blinded, randomized, crossover, diet-controlled studies and assessing cholesterol and triacylglycerol absorption based on results from TRL fractions versus plasma. Although assessments of cholesterol and triacylglycerol responses in TRL fractions were secondary measurements, retrospective power calculations support both studies having adequate sample size. It is important to note that these results come from acute feeding trials and postprandial changes in total cholesterol content in TRL fractions includes both dietary and biliary cholesterol. Estimates indicate that biliary cholesterol contributes about 75–85% of intestinal cholesterol content [4]. The experimental design and methods we used preclude distinguishing between dietary and biliary cholesterol in TRL fractions and the greater quantity of biliary cholesterol in the intestinal lumen may affect the accuracy of dietary cholesterol absorption assessments. A priori, we chose to use the AUC results as the foundation for interpreting the study. The AUC-based result that cholesterol from whole eggs is not well absorbed is also shown at each postprandial time point.

## 5. Conclusions

In conclusion, results from these two randomized controlled acute feeding studies indicate that dietary cholesterol contained in whole egg is not well absorbed and does not increase plasma total cholesterol concentration. These findings provide a mechanism to help explain why dietary cholesterol intake may not affect long-term plasma total cholesterol control.

## Supplementary Materials

The following are available online at http://www.mdpi.com/2072-6643/10/9/1272/s1, Figure S1: Baseline-corrected total cholesterol (A) and triacylglycerol (B) content in the TRL fraction in study 1, Figure S2: Baseline-corrected total cholesterol (A) and triacylglycerol (B) content in the TRL fraction in study 2, Figure S3: Baseline-corrected total cholesterol (A) and triacylglycerol (B) content in plasma in study 1, Figure S4: Baseline-corrected total cholesterol (A) and triacylglycerol (B) content in plasma in study 2.
