# Supplementary material for: Dietary Cholesterol Contained in Whole Eggs Is Not Well Absorbed and Does Not Acutely Affect Plasma Total Cholesterol Concentration in Men and Women: Results from 2 Randomized Controlled Crossover Studies
Source: Nutrients. 2018 Sep 9;10(9):1272. doi: 10.3390/nu10091272 (PMC6165023; doi:10.3390/nu10091272)

Supplemental Figure 1. Baseline-corrected total cholesterol (A) and triacylglycerol (B) content in the TRL fraction in study 1. Values are lsmean ± SE and different superscript letters indicate statistical differences among no egg, 75 g CWE, and 150 g CWE. CWE, cooked whole egg; TRL, triacylglycerol-rich lipoprotein.

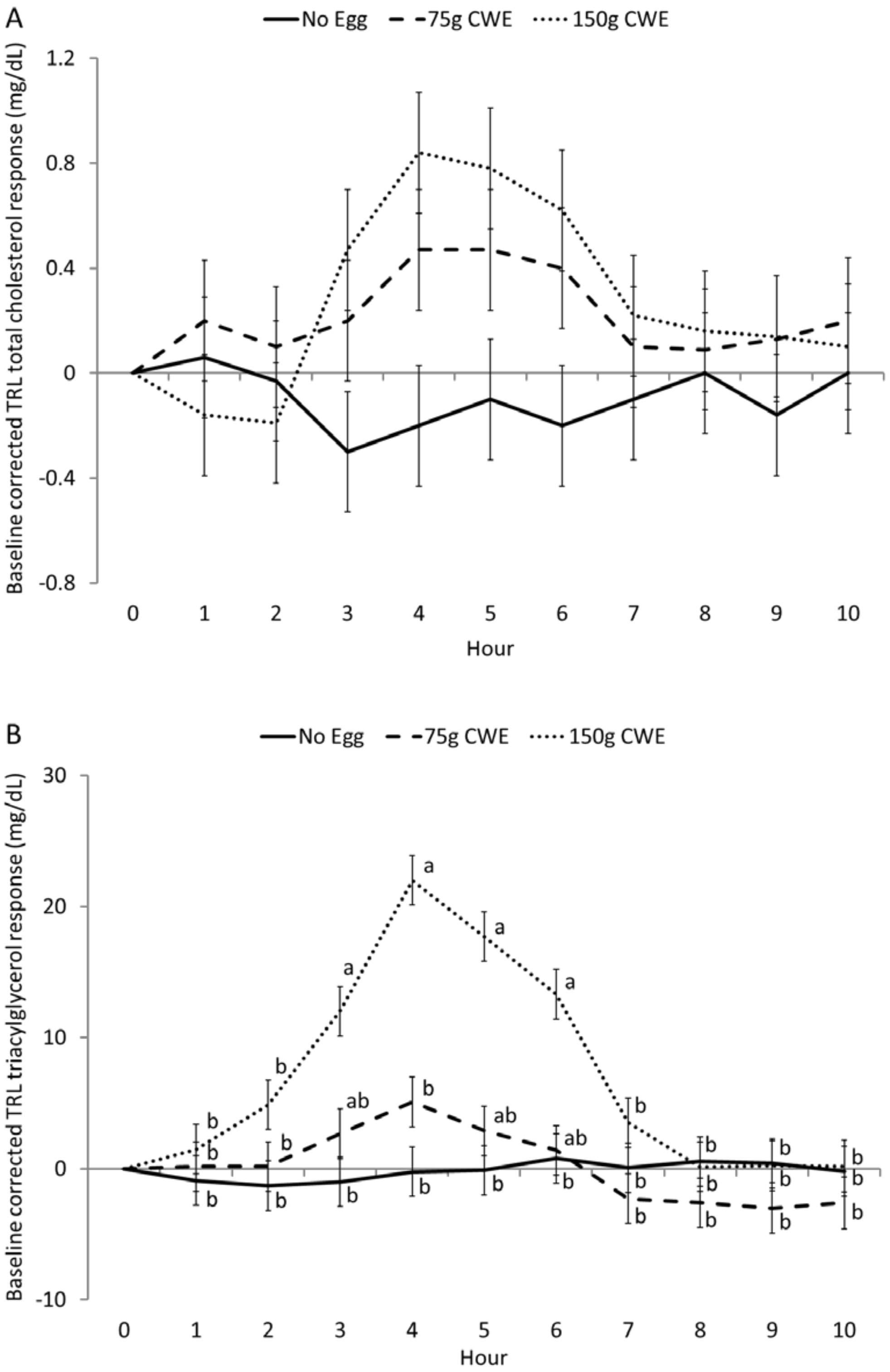

Supplemental Figure 2. Baseline-corrected total cholesterol (A) and triacylglycerol (B) content in the TRL fraction in study 2. Values are lsmean ± SE and different superscript letters indicate statistical differences between no egg and 100 g CWE. CWE, cooked whole egg; TRL, triacylglycerol-rich lipoprotein.

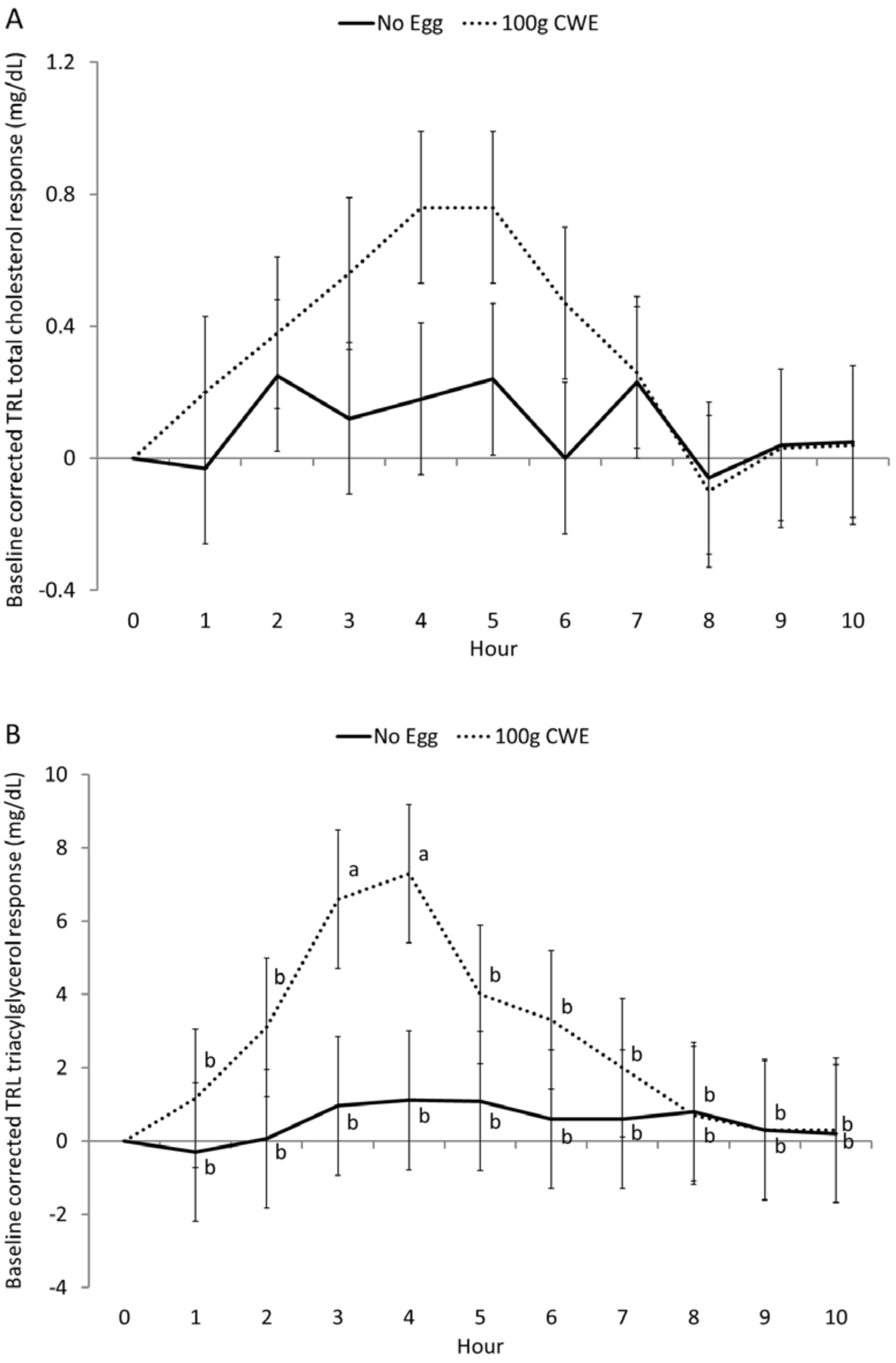

Supplemental Figure 3. Baseline-corrected total cholesterol (A) and triacylglycerol (B) content in plasma in study 1. Values are lsmean ± SE and different superscript letters indicate statistical differences among no egg, 75 g CWE, and 150 g CWE. CWE, cooked whole egg.

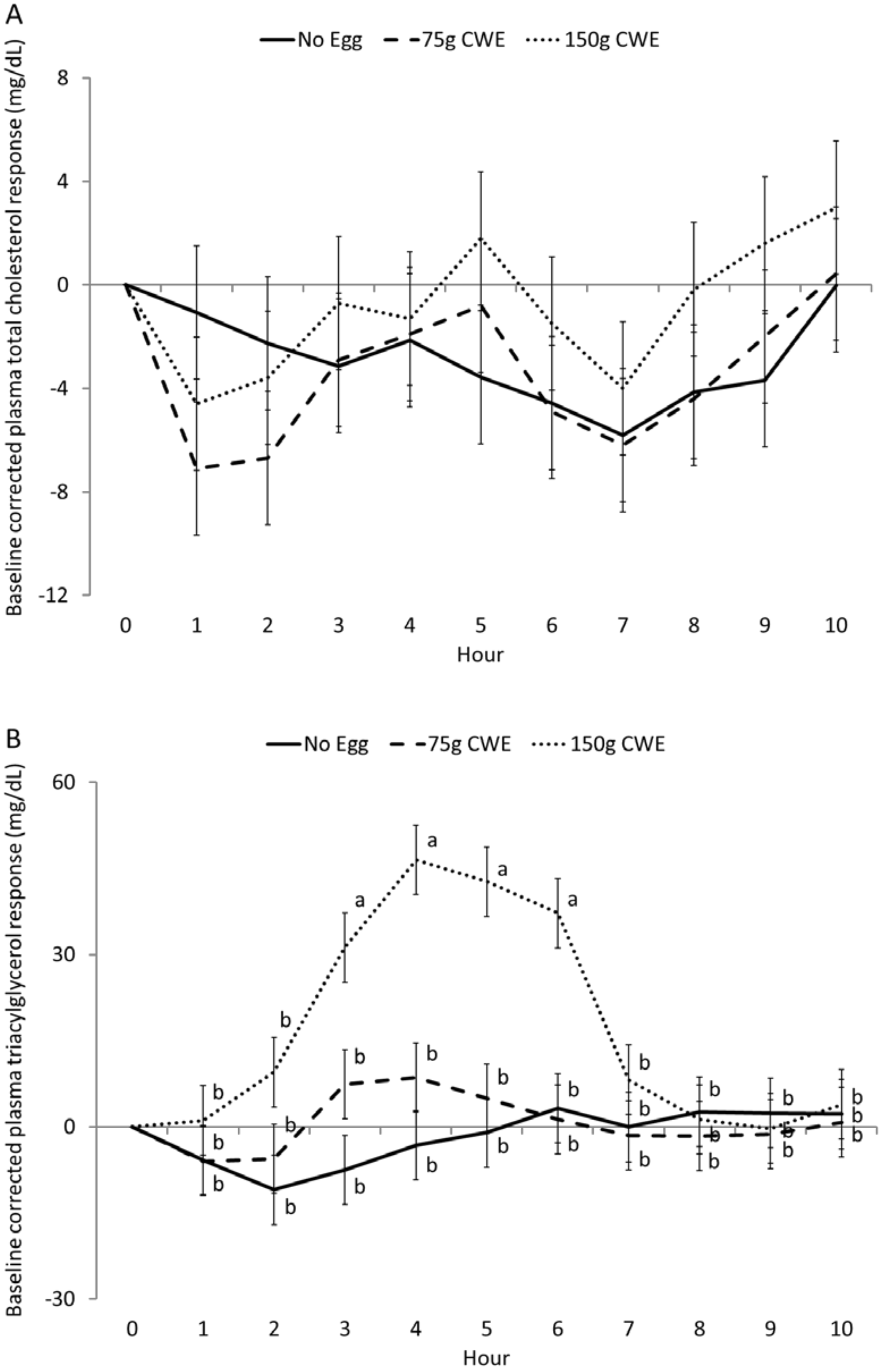

Supplemental Figure 4. Baseline-corrected total cholesterol (A) and triacylglycerol (B) content in plasma in study 2. Values are lsmean ± SE and different superscript letters indicate statistical differences between no egg and 100 g CWE treatments. CWE, cooked whole egg.

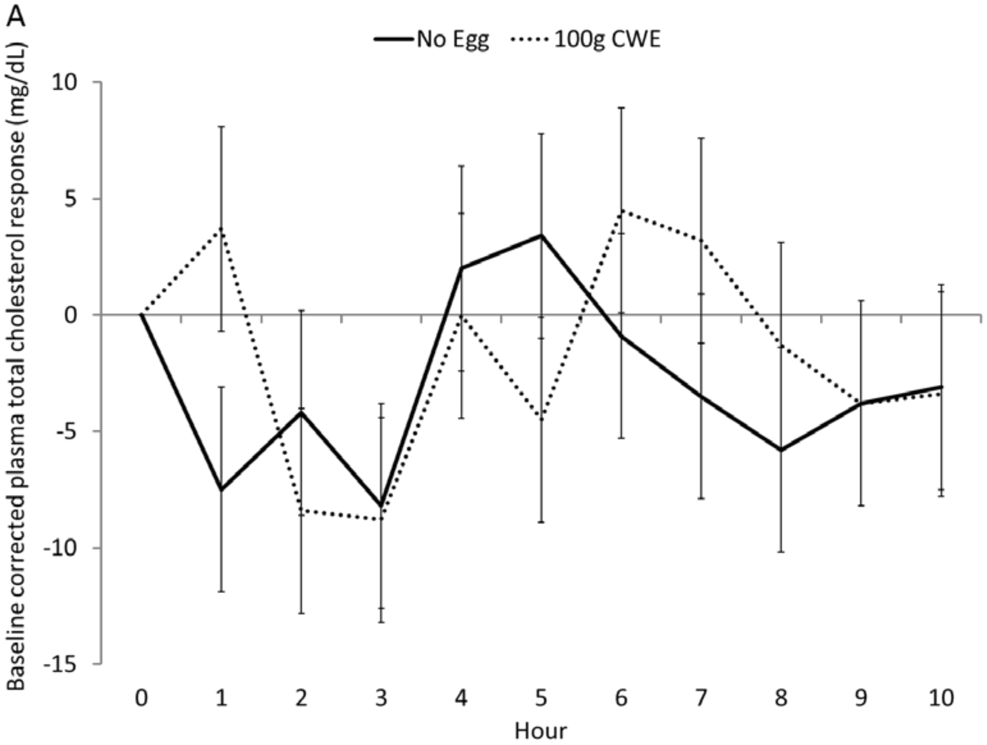

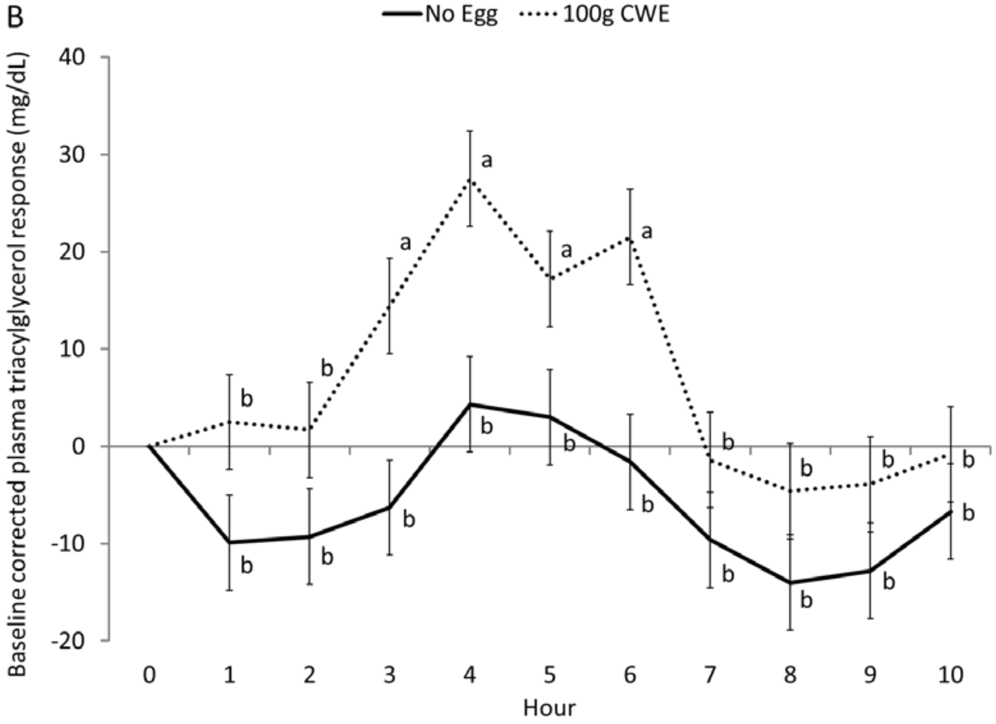

Supplement: Supplementary file 1 [file nutrients-10-01272-s001.pdf]
